# The development and evaluation of single cell suspension from wheat and barley as a model system; a first step towards functional genomics application

**DOI:** 10.1186/1471-2229-10-239

**Published:** 2010-11-05

**Authors:** Jing Dong, Steve Bowra, Eva Vincze

**Affiliations:** 1 Dept. of Genetics and Biotechnology, Faculty of Agricultural Sciences, Aarhus University, Research Centre Flakkebjerg, DK-4200 Slagelse, Denmark; 2 Agronomy Dept., Huajiachi Campus, Zhejiang University, Hangzhou 310029, China; 3 Verzyme (UK) Ltd., Plas Gogerddan, Aberystwyth, Wales SY23 3EB, UK

## Abstract

**Background:**

The overall research objective was to develop single cell plant cultures as a model system to facilitate functional genomics of monocots, in particular wheat and barley. The essential first step towards achieving the stated objective was the development of a robust, viable single cell suspension culture from both species.

**Results:**

We established growth conditions to allow routine culturing of somatic cells in 24 well microtiter plate format. Evaluation of the wheat and barley cell suspension as model cell system is a multi step process. As an initial step in the evaluation procedure we chose to study the impact of selected abiotic stress elicitors at the physiological, biochemical and molecular level. We report the results of osmotic stress imposed by NaCl and PEG. As proline is an important osmoprotectant of the cereal cells, colorimetric assay for proline detection was developed for small volumes (200 μl). We performed RT-PCR experiments to study the change in the expression of the genes encoding Δ^1^-pyrroline-5-carboxylate synthetase (P5CS) and Δ^1^-pyrroline-5-carboxylate reductase (PC5R) in response to abiotic stress.

**Conclusions:**

We found differences between the wheat and barley suspension cultures, barley being more tolerant to the applied osmotic stresses. We suggested a model to explain the obtained differences in stress tolerance between the two species. The suspension cell cultures have proven useful for determining changes in proline concentration and expression level of genes (P5CS, P5CR) under various treatments and we suggest that the cells can be used as a model host system to study gene expression and regulation in monocots.

## Background

Plant cell culture has a very long history. It was in 1902, when German botanist Gottlieb Haberlandt published the article "Cultuversuche mit isolierten Pflanzenzellen" which described his vision of the totipotency of plant cells [1]. Since then plant cell suspension cultures have been used not only for clonal plant propagation but also to investigate the physiological, biochemical, and molecular aspects of various cellular functions. For instance, isolated plant cells have been extensively used to study photosynthesis [2], ion transport [3,4], secondary metabolite production [5], cell growth and differentiation [6] and programmed cell death [7]. Recently *Arabidopsis *and *Zinnia *cell suspension cultures were reported to be used in multiple gene detection and genome wide gene expression studies [8-10].

However, while cell suspensions have long been recognised as a useful tool, the direct relevance of the physiology and biochemistry of a cell line to that of the whole plant remains a subject of considerable debate [11-14]. Currently the greater proportion of work with plant cell suspensions relates to dicot 'model' plants i.e. Arabidopsis and tobacco, therefore in the light of this we have chosen to develop single cell plant suspension cultures of wheat and barley as a model host system.

Increasingly it is being recognised that, under the appropriate conditions plant cell suspensions i.e. culturing cell in small multi-well plates could become an ideal platform to assist plant functional genomics by aligning with high-throughput (HTP) technologies, which have the capability to provide a global perspective on gene expression and gene product accumulation/interaction. Coupling HTP technologies with culturing cell in small multi-well format would serve two purposes; it would provide a vehicle whereby the effect of different elicitors on target gene expression can be assessed and the data acquired from somatic plant cells in culture under a range of environmental conditions can be compared with the expression profile *in planta*.

The aims of our study were a) to develop a robust, viable cereal single cell suspension system that can be maintained in a multiwall format and b) validate the platform by investigating the impact of abiotic stress on selected gene expression in order to draw comparisons between the in-vitro model system and *in planta*. Many plants when subject to different abiotic stress conditions such as salt and drought stress accumulate compatible solutes such as proline and glycine betaine [15,16]. Proline is an amino acid which dominates barley storage proteins but also performs an important function as a protective compatible osmolyte, scavenging free radicals [17-19]. Although proline can be synthesized from either glutamate or ornithine under osmotic stress, glutamate is the primary route of de novo synthesis [20]. The first two steps are catalysed by Δ^1^-pyrroline-5-carboxylate synthetase (P5CS), a bifunctional enzyme with apparent activities of γ-glutamyl kinase (γ-GK) and glutamic acid-5-semialdehyde (GSA)-dehydrogenase [21]. During the process glutamate is phosphorylated by γ-GK to γ-glutamyl phosphate, which is then reduced to GSA by GSA dehydrogenase. GSA spontaneously cyclyzes to Δ^1^-pyrroline-5-carboxylate (P5C), which is reduced to proline by Δ^1^-pyrroline-5-carboxylate reductase (P5CR) in the final step. The rate limiting step in this pathway is represented by the γ-glutamyl kinase activity of P5CS, which is thought to be sensitive to feedback inhibition by relatively low levels of proline [22]. Given the documented whole plant response of proline to abiotic stress and the accumulation of storage proteins rich in proline we have chosen to utilise P5CS and P5CR genes as molecular indicators of the cell suspension's response to abiotic stress.

In this study we report the development the single cell suspension cultures of wheat and barley and demonstrated the utility of the cells in a multi-well format as a step forward to using the platform in high-throughput technologies. The response of the cell lines to abiotic stress were characterised at the molecular and biochemical level enabling comparison with *in planta *response. These experiments were performed as first step toward validating the cell suspensions of barley and wheat as model systems for functional genomics.

## Methods

### Origin of the tissue cultures

Wheat (PC 998) and barley (PC 1163) callus lines were obtained from the Plant Cell Culture Collection of DSMZ (Sammlung von Mikroorganismen und Zellkulturen GmbH, Braunschweig, Germany). The wheat line PC 998 derived from *Triticum aestivum *L. emend. Fiori et Paol, while the barley line PC 1163 was derived from *Hordeum vulgare *L. Sommergerste Salome.

### Development of single cell suspensions of wheat and barley

**Figure 1:**
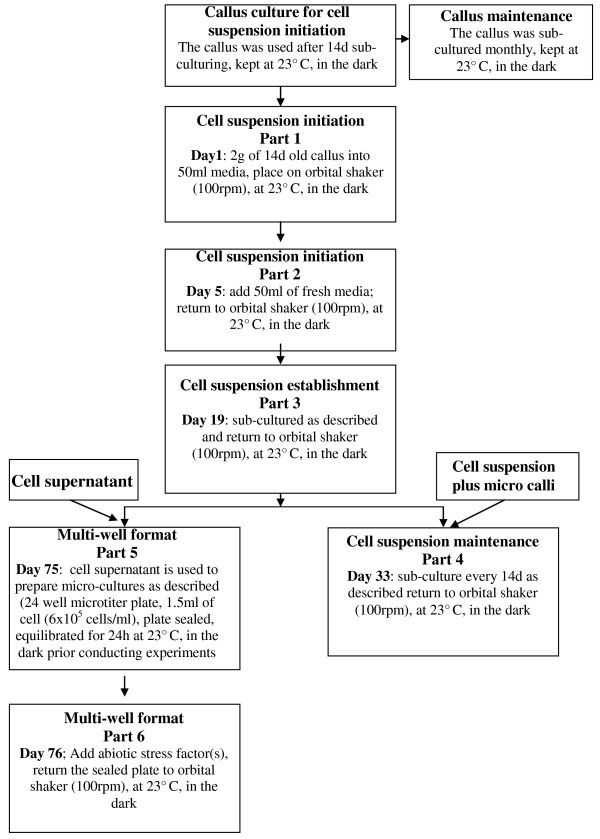
**Chart flow of the initiation and the maintenance of the wheat and barley single cell suspension cultures**. Detailed description can be found in the Method section.

The wheat and barley cell lines were maintained as a callus in the dark at 24°C on media described in Table 1 and sub-cultured monthly by transferring one third of the callus to fresh plate. The development and maintenance of the single suspension culture are described in the flow chart (Figure 1). The optimal stage to initiate the cell suspension was 14 days after sub-culturing the callus. The single cell suspension was started with approximately 2 g of callus transferred under aseptic conditions into 50 ml of media (Table 1) in a 250 ml Erlenmeyer flask where it was broken up with gently shaking. The flask was shaken at 100 rpm using an orbital shaker (Certomat SII, Sartorius) and maintained at 23°C in the dark for 5 days before the media was topped up with 50 ml of fresh media. After an additional 12 days of shaking the original crude suspension was sub-cultured. It was found during preliminary experiments that using a 1 ml Gilson pipette, even with a tip cut to create a larger aperture, did not enable the transfer of enough micro callus and after several cell passages the suspension began to loose viability. Therefore, to overcome the lose of cell viability with time, the sub-culture step was optimised and is described as follows: Prior to sub-culturing the 250 ml Erlenmeyer flask with the single cells and micro callus population was allowed to stand. The micro callus settled out of solution, leaving a complex mixture of dead and viable cells in the suspension. Two thirds of the supernatant was gentle decanted from the flask leaving one third of the supernatant One third of the residual 'mixture', made up of single cells, plus the micro callus, was poured from the flask and used to inoculate 100 ml fresh media in a 250 ml Erlenmeyer flask. The freshly sub-cultured material was returned to the shaker (100 rpm) and left at 23°C in the dark for 12 days before repeating the sub-culturing procedure. After a further 3 to 4 passages, the cell suspension contained an average of 6 × 10^-5 ^cell/ml with 65% viable single cells in the supernatant. The cell suspension cultures maintained a minimum 65% single cell viability for 6 months when the cell suspension were sub-cultured as described above. However after 6 months, the suspension cultures started to loose the ability to produce single cells and therefore the suspension was re started.

**Table 1:** Media composition for the wheat and barley tissue and suspension cultures.

Component	Medium B5	Medium HV
	**mg/l**	**mg/l**

NaH_2_PO_4 _× H_2_O	172	

KH_2_PO_4_		34

CaCl_2 _× 2 H_2_O	150	88

(NH_4_)_2_SO_4_	134	

NH_4_NO_3_		1650

MgSO_4 _× 7 H_2_O	250	370

KNO_3_	2500	1900

FeSO_4 _× 7 H_2_O	25.6	11.12

Na_2_EDTA × 2 H_2_O	34.27	15.72

KJ	0.75	0.166

MnSO_4 _× H_2_O	10	4.46

H3BO_3_	3	1.24

ZnSO_4 _× 7 H_2_O	3	1.72

Na_2_MoO_4 _× 2 H_2_O	0.25	0.05

CuSO_4 _× 5 H_2_O	0.25	0.005

CoCl_2 _× 6 H_2_O	0.25	0.005

Nicotinic acid	1	1

Thiamine hydrochloride	10	10

Pyridoxal hydrochloride	1	1

myo-Inositol	100	100

2,4-Dichlorophenoxyacetic acid	2	2

Sucrose	20000	30000

(Agar)	(8000)	(8000)

	pH = 5.5	pH = 6.0

(Sammlung von Mikroorganismen und Zellkulturen GmbH, Braunschweig, Germany).

Sterilized at 120°C for 30 minutes

### Evaluation and optimisation of fluorescein diacetate (FDA) assay for testing cell viability

The fluorescein diacetate (FDA) assay described by Rotman [23] was modified and conducted as follows; the stock solution (1 mg FDA ml^-1 ^in acetone, kept at -20°C) was diluted 10× before use with the appropriate plant media (Table 1). Kinetics of the FDA reaction where carried out using a final substrate concentration of 100 ng, 200 ng and 300 ng in total volume of 50 μl. Total fluorescence of the cell population was measured using a microtitre plate reader (Synergy2, Bioteck) set up in the fluorescence mode with excitation wavelength set to 485 nm and emission wave length set to 530 nm. The fluorochromatic reaction of single cells was visualised using a Zeiss Axioplan 2 fluorescent microscope. The total cells in the sample were estimated by using a haematocytometer. The cell viability was reported as a percentage of living cells in the total cell population.

### Maintaining the single cell suspension cultures in multi well format

To prepare the single cell suspension in a multi-well the cell suspension was first allowed to stand before two thirds of the supernatant was decanted as described above. The decanted supernatant was used as the source of single cells for the evaluation of the potential of a multi-well cell culture format. To establish the viability of these cells in 24 well microtiter plate formats, one and half ml of cells at a concentration of 6 × 10^-5 ^cells/ml was added to the wells using 1 ml micropipette with a cut tips, the lid of the microtiter plate was replaced and sealed with parafilm. The sealed microtiter plate was placed at 23°C in the dark, on an orbital shaker at 75 rpm to achieve gentle agitation to equilibrate prior to conducting the abiotic stress experiments The cell viability was determined using fluorescein diacetate (FDA) assay (see above) over a 10 day period.

### P5CR protein detection in the wheat and barley suspension cultures

Salt soluble proteins isolation: 2 ml of the cell suspension culture was centrifuged and the supernatant discarded. Three hundred and fifty mg fresh cells were re-suspended in 150 μl extraction buffer (0.15 M potassium phosphate buffer, pH 8.0, 5 mM dithiothreitol). The proteins were extracted for 1 hour at 20°C (room temperature) with vigorous shaking and the centrifuged at 1000 g (~3900 rpm) for 8 minutes. Proteins were resolved on a 4 to 12% NuPAGE gradient gel (Invitrogen Ltd) according the company instruction. Electrophoretic transfer of proteins from sodium dodecylsulfate-polyacrylamide gels to nitrocellulose membrane was performed using blotting buffer (20% methanol, 25 mM Tris buffer pH 8.8, 0.05% SDS) at 48 mA for one and half hour [24].

The blot was blocked using 5 M QuickBlock kit (GenScript Corporation). The primary antibody was produced by GenScript: A peptide containing 14 amino acids common between wheat and barley was chosen from the aligned wheat and barley P5CR sequences (P5CR: *T. aestivum *GenBank:AAW82908 and *H. vulgare *GenBank:AY177684), synthesised and polyclonal peptide antibody was produced. Five μl primary P5CR peptide antibody was added to 4 ml TBST buffer (20 mM Tris pH 7.5, 150 mM NaCl, 0.05% Tween 20) with 1 ml 2% casein (in PBS with 3 mM sodium azide). Immunobloting was performed in a sealed plastic bag gently shaking for 60 min at room temperature. The membrane was rinsed and incubated in 20 ml TBST for 10 min. Anti rabbit IgG alkaline phosphatase conjugate was used as a secondary antibody at a dilution of 10 μl in 25 ml TBST (Promega) and incubated at RT with gently 20 min. The membrane was further rinsed in 20 ml TBST for 10 min followed by a deionised water rinse. The alkaline phosphatase assay was conducted using Sigma Fast BCIP/NBT according to the manufactures protocol) and stopped by washing the membrane in water after 40 min.

### Osmotic stress experiments

The single cells suspensions derived from the wheat (PC 998) and barley (PC 1163) callus cultures were used to study the impact of abiotic stress. The experiments were conducted with suspensions with cell population density of 6 × 10^-5 ^cells/ml at 60-65% viability. One and half ml aliquots of the cell suspension were dispensed into 24 well microtiter plates and allowed to equilibrate for 24 h prior to the application of 0, 50, 100 mM NaCl, or 5% PEG 6000. The volumes were adjusted by adding sterile distilled water to ensure that the final volumes of the cell suspension were the same across the treatments. The experiments were performed in triplicates. Cells were sampled at 0 h, 2 h and 24 h after the applications of the osmotic stress and cell viability, proline content and expression of the tubulin, P5CS and P5CR genes were measured.

### Determination of proline content

To measure free proline, a micro-assay was developed where 200 μl of plant cell suspension was homogenised in 3 ml of 3% sulfosalicylic acid in BIO101 Savant centrifuge (20 seconds, at speed 5.5) with a ceramic ball in the 2 ml screw cap eppendorf tubes. Free Proline was measured in the homogenised plant cell material as described by Bates et al. [25] using L-Proline as a standard.

### RNA extraction; cDNA synthesis and RT PCR

Two hundred microlitres of cells were harvested placed in a 1.5 ml eppendorf and centrifuged at 12000 rpm for 1 min (Eppendorf centrifuge 5417C). The supernatant was removed and the cell pellets were frozen in liquid nitrogen and stored at -80°C. Total RNA was isolated using the FastRNA^® ^Pro Green Kit according to the manufacturer's protocol. RNA was quantified using 2100 Agilent Bioanalyzer. First strand cDNA synthesis was carried out using 2 μg of total RNA according to the manufacturer instruction (Invitrogen GmbH, Karlsruhe, Germany). The cDNA synthesis reaction was terminated by increasing the 40 μl reaction mix to 200 μl with addition of sterile distilled water.

In order to design primers common to wheat and barley tubulin, P5CR and P5CS, the following accessions were recovered from the GenBank (tubulin: *T. aestivum *tubulin 3A GenBank:DQ435663, *H. vulgare *α-tubulin 3 GenBank:AJ132399; P5CS *T. aestivum *GenBank:AB 193551 and *H. vulgare *GenBank:AK249154; P5CR: *T. aestivum *GenBank:AAW82908 and *H. vulgare *GenBank:AY177684), and aligned Table 2.

**Table 2:** Origin of the genes and the primers for the RT-PCR experiments.

Name	Primers
Tubulin	F2 5' CCTCATCACCGTCCTCGCC 3' R2 5' TGATCTCAGCTGAGAAGGC 3'

P5CS	F1 5' CGTGAAGCGCATCATAATCA 3' R1 5' ATCAGCCCACTCTGACCAAC 3'

P5CR	F1 5' AACGAACCCTTCTCGAGCTCATG 3' R1 5' AGCAGCATCAGTGATGTGTCTAG 3'

For the primer design NCBI gene bank entries were used (tubulin: *T. aestivum *tubulin 3A DQ435663, *H. vulgare *α-tubulin 3 AJ132399; P5CS *T. aestivum *AB 193551 and *H. vulgare *AK249154; P5CR: *T. aestivum *AAW82908 and *H. vulgare *AY177684).

PCR was carried out using 2 μl of first-strand cDNA by adding 5.0 μl of Advantage 10× PCR buffer, 0.5 μl of 20 mM dNTP, 1.5 μl 5 μM of sense and antisense primers, 0.5 μl 10U of Advantage 2 Taq (Clontech), 40 μl H_2_O. Table 2 contains the information about the origin of the genes mentioned in the study and the designed primers sets for the RT-PCR experiments. The thermal profile for the PCR was 95°C for 3 min, followed by 30 cycles of 94°C for 30 s, 60°C for 30 s, 72°C for 45 s. The final elongation was performed at 72°C for 7 min. The resulting PCR products were resolved using agarose gel electrophoresis. The gels were scanned using BioRad Molecular Imager FX and image analysis performed using ImageJ software http://rsb.info.nih.gov/ij. The experiments were repeated twice giving very similar pattern of responses.

### Statistical evaluation

Data on cell viability and proline content were analysed with a repeated measures ANOVA, using post-hoc Tukey tests where relevant.

## Results

### Development of single cell suspensions of wheat and barley

**Figure 2:**
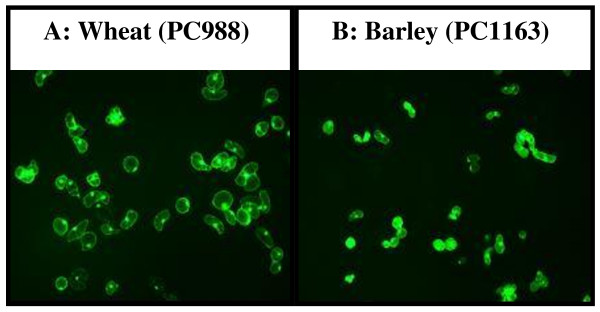
**Viability and morphology of the wheat and barley cells from the suspension cultures**. Zeiss Axioplan 2 fluorescent microscope was used for visualising the fluorescein diacetate (FDA) treated cells. Data are the means of four independent experiments, and bars represent ± SE; n = 4.

Plant cell culture conditions developed to support the creation of viable single cell suspension are illustrated in Figure 1. It was empirically established that the callus, which was vigorously growing 14 days after sub-culturing, was friable and at the optimal morphological and physiological stage to initiate the cell suspension. In our case, the optimal procedure for establishing the single cell suspension was to start with approximately 2 g fresh weight of callus. After 12 days, the original crude suspension culture contained a range of micro callus and single cells. Analysis of the cell viability indicated that after the first 12 days 30% of the single cells were viable. The procedure for sub-culturing the suspension was empirically optimised with a view to increasing the number of viable single cells in the suspension population. Conventionally, cell cultures are sub-cultured by inoculating 50 ml of new fresh media with 10 ml of culture. It was found that using a pipette excluded micro callus, and after several cell passages the suspension began to loose viability. Experiments were carried out to evaluate the impact of transferring 'nurse' cells in the form of micro callus, the results led to the optimised practise described in the Methods (Figure 1). Adopting the procedure as described fostered vigorous cell growth and an increase in the percentage of viable cells. After a further 3 to 4 passages the cell suspension contained 6 × 10^-5 ^cell/ml with approx. 65% viable single cells (Figure 2), which can be maintained for 6 months with sub-culturing every 14 days.

### Evaluation of fluorescein diacetate (FDA) assay for testing cell viability

**Figure 3:**
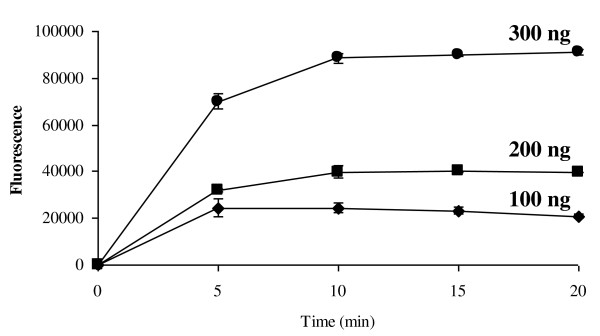
**Production of fluorescein by wheat suspension culture**. Effect of the fluorescein diacetate (FDA) concentration on the uptake and production of fluorescein by wheat cells.

Rotman [23] reported the utility of FDA, a non polar compound, as marker for mammalian cell viability. The model proposed was that cell membrane is permeable to the non-polar substrate and less permeable to the polar product fluorescein. The non polar compound is a substrate that can be enzymatically cleaved and the fluorescent product which is retained within the cell produces a bright green fluorescent image. Preliminary experiments were performed to evaluate the utility of FDA assay with wheat and barley cells were conducted and the cell fluorescence visualised using a Zeiss Axioplan 2 fluorescent microscope the results are shown in Figure 2. A series of experiments were performed to optimise and evaluate the kinetics of the reaction in wheat and barleys (Figure 3). The results showed that the reaction begins instantly reaching a maximum at approximately 10 mins for the highest concentration studied in wheat. Barley cells produced the same curves (data not shown). The initial rate of reaction appears to be substrate dependent and for the highest substrate the rate of change in fluorescence was 15000/min. From the results it was concluded that final concentration of 200 ng FDA was sufficient to visualise the viable cells after 10 min incubation therefore this concentration was used in the further experiments.

### P5CR protein accumulation in the wheat and barley suspension cultures

**Figure 4:**
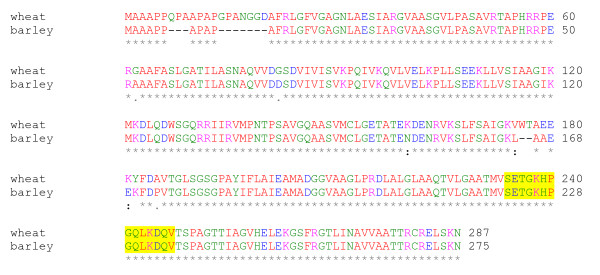
**The predicted P5CR protein sequence comparison from wheat and barley**. The wheat protein sequence is from GenBank (*Triticum aestivum *GenBank:AAW82908) and *Hordeum vulgare *GenBank:AY177684). The comparison was performed with CLUSTAL W (1.83) multiple sequence alignment program. The highlighted region is the peptide, which was used for the antibody production.

**Figure 5:**
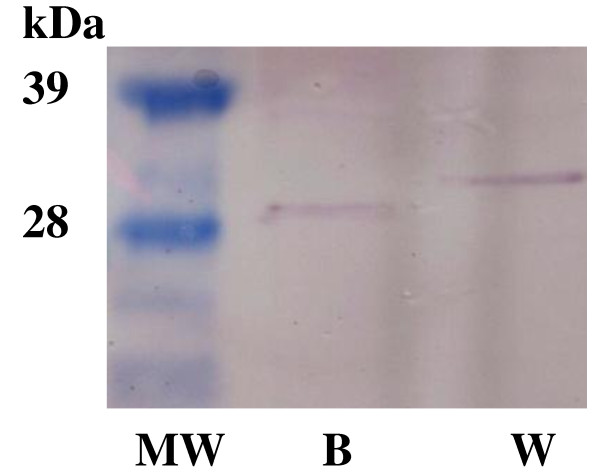
**Western blots of the suspension culture**. P5CR protein identification from the salt soluble fraction of the suspension cultures were performed by immuno blotting: with polyclonal P5CR peptide antibody. MW: high protein molecular weight marker (Invitrogen Inc); B: barley and W: wheat.

Further to reinforce the viability of the suspension cultures, Western hybridisation experiments were performed to show that the P5CR proteins accumulated in both wheat and barley cell suspension cultures. Polyclonal peptide antibody was produced as described in the Methods using the established similarities between wheat and barley P5CR sequences (Figure 4). The predicted MW for the wheat P5CR gene is 29.3 kDa, while it is 28.2 kDa for the barley protein. The Western experiments confirmed the presence of the P5CR proteins and the MW differences (Figure 5).

### Evaluation of multi-well cell culture format

As stated the objective was to establish conditions where single cell suspension could be handled and efficiently manipulated in a multi-well format, which in turns could be aligned with high-throughput applications. To achieve this, a 24 well format was adopted. To prepare the microtitre plate, 1.5 ml of cells at a concentration of 6 × 10^-5 ^cell/ml was added to individual wells using a 1 ml pipette with cut tips. The cell viability was determined as described over 10 days period. During the course of the experiment, the number of viable cells expressed as a percentage of the total cell population did not change significantly (data not shown). The lack of change in cell viability over 10 days lead to the conclusion that the cells maintain in a 24 well format were stable and were therefore a convenient 'tool' for further experiments.

### Impact of NaCl and PEG mediated abiotic stress on wheat and barley cell viability

**Figure 6:**
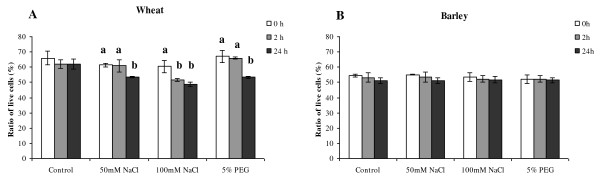
**Changes in cell viability under osmotic stresses**. Cell viability was expressed as a percentage of the number of cells that remained alive in media supplemented with 50 mM NaCl, 100 mM NaCl, or 5% PEG 6000 with respect to time. A) Wheat (PC998) B) Barley (PC1163). Data are the means of three independent experiments, and bars represent ± SE; n = 3. Values marked with different letters are significantly different from the respective control at P <0.05 as detected by a repeated measures ANOVA.

Both wheat and barley single cells were subject to 50 mM, 100 mM NaCl and 5% PEG stress treatments. The cell viability was estimated at time 0 h, 2 h and 24 h using the FDA assay. The results, summarized in Figure 6, were reported as a percentage of living cells within the total cell population.

The single cell suspension of wheat under control conditions (no treatment) did not exhibit substantial differences in the percentage of living cells over the duration of the experiment. After 2 hours the percentage cell viability in the presence of 100 mM NaCl appears to have decreased while the cell viability treated with 5% PEG appears to show a slight increase (Figure 6A). However after 24 h the percentages of viable cell had decreased with all three treatments (Figure 6A). The percentages of viable cell had decreased significantly (p < 0.05) with time in all three treatments and the orders were 0 h = 2 h > 24 h in case of 50 mM NaCl and 5% PEG treatments, while in the 100 mM NaCl treatment the order was 0 h > 2 h = 24 h. More specifically after 24 hours the ratio of living viable cells to dead cells as a percentage within the total cell population, in the presence of 50 mM NaCl decreased to 87.0% relative to the control. In the presence of 100 mM NaCl the percentage of viable cells dropped to 80.6%. The PEG treatment also resulted in 20% reduction in the number of viable cells (Figure 6A).

The barley tissue culture line PC1163 was tested under the same stress conditions. The barley cells survival rates did not change significantly (repeated measures ANOVA, Tukey post-hoc comparisons, p = 0.17-1.00) in any of the treatments (Figure 6B).

### Accumulations of proline in wheat and barley cells in response to NaCl and PEG stress

**Figure 7:**
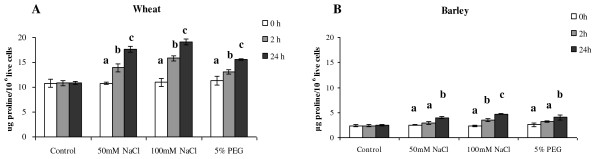
**Changes in proline content of the cells under osmotic stresses**. Proline content of live cells in media supplemented with 50 m M NaCl, 100 mM NaCl, or 5% PEG with respect to time. A) Wheat (PC998); B) Barley (PC1163). Data are the means of three independent experiments, and bars represent ± SE; n = 3. Values marked with different letters are significantly different from the respective control at P <0.05 as detected by a repeated measures ANOVA.

On exposure to environmental stress conditions, such as salt and drought stress, many plants accumulate compatible solutes, for example proline and glycine betaine, which are thought to offer physiological and biochemical protection enabling the organism to tolerate transient and in some cases sustained exposure to environmental stress. Given these observations, we measured the amount of proline in wheat and barley cells under abiotic stress condition (Figure 7).

The results indicated that the wheat line responded to 50 mM, 100 mM NaCl and 5% PEG treatments by accumulating significantly increased amounts of proline with time (Figure 7A). In all treatments the measured amount of proline was significantly different (p < 0.05) along time in the order 0 h<2 h < 24 h. In the cells, which remained alive, the increase in proline levels at 2 hours in the presence of 50 mM, 100 mM NaCl and 5% PEG were 28.4%, 46.2% and 20.5, respectively while at 24 h the increases in the percentage of proline levels were 62.3% 75.8% and 43.6% (Figure 7A).

In contrast to the survival rates of the barley cells, which did not change with the treatments, the proline level appears to increase significantly in both salt treatments (50 mM and 100 mM NaCl) and in the presence of 5% PEG after 24 h (Figure 7B). The increases were markedly different even after 2 h (Figure 7B). The measured amounts of proline were significantly different (p < 0.05) in the order 0 h<2 h < 24 h in case the 100 mM NaCl treatment, while in the 50 mM NaCl and 5% PEG treatments, the order was 0 h = 2 h < 24 h. In the cells, which remained alive, the measured increases in proline levels in the presence of 50 mM, 100 mM NaCl and 5%PEG at 2 h were 21.7%, 47.9% and 33.3%, respectively, while at 24 h the increases in the percentage of proline levels were 58.9% 87.7% and 61.8% (Figure 7B).

### Steady state level of mRNA for genes encoding proline biosynthesis enzymes under abiotic stress

The proline levels were shown to increase in response to the abiotic stress treatment. Using RT PCR normalised against tubulin, the impact of the treatment on the steady state level of P5CS and P5CR transcripts, the two central genes involved in proline biosynthesis, was determined. The experiments were repeated and gel imagines were evaluated using ImageJ software. Both experiments produced the same results in terms of response trend, however technical variation with the image analysis did not allow the both data sets to be combined at quantitative level; therefore we have reported one experimental data set.

**Figure 8:**
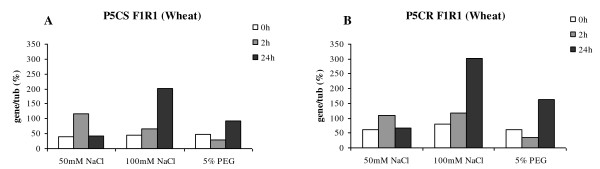
**Wheat gene expression studies under osmotic stresses**. The RT-PCR expression analysis of the genes encoding wheat P5CS and P5CR were performed from cells subject to abiotic stress with respect to time. The graphs were generated by normalizing gene of interest expression against tubulin using the image analysis software ImageJ.

Analysis of the steady state levels of the mRNA from wheat cell in the presence of 100 mM NaCl suggests that the treatment induced the accumulation of the P5CS transcript (Figure 8A). After 24 h the steady state level of the P5CS transcript was 5 times higher than the control. However, under the 50 mM NaCl treatment the impact was less clear. After 2 h the steady state level of P5CS transcript appeared to increase, however, at the 24 h time point there appears to be no increase in the transcript level relative to the control, this remains unexplained. Analysis of the steady state level of the P5CR transcript revealed that although the actual transcript level was one third higher compared to P5CS, similar pattern of transcript accumulation could be observed over time (Figure 8B).

**Figure 9:**
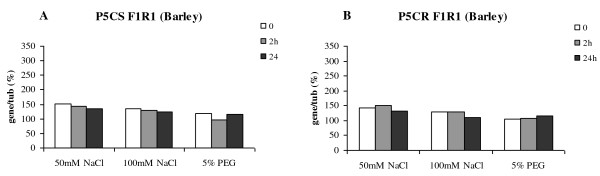
**Barley gene expression studies under osmotic stresses**. The RT-PCR expression analysis of the genes encoding barley P5CS and P5CR from cells subject to abiotic stress with respect to time. The graphs were generated by normalizing expression of gene of interest against tubulin using the image analysis software ImageJ.

The analysis of the mRNA for P5CS and P5CR genes from the barley single cell cultures (Figure 9A and 9B) revealed that the steady state level of the transcripts did not change in response to either treatments or time. Although within the error of the experiment, paradoxically it could be suggested the level of transcript accumulation decreased under the salt treatment.

## Discussion

The overall research objective was to develop single cell plant cultures as a model host system to facilitate functional genomics of monocots, in particular wheat and barley. Evaluation of the wheat and barley model plant cell suspension system is a multi step process. The essential first step towards achieving the stated objective was the development of a robust, viable single cell suspension culture that can be maintained in a multi-well format. The established growth conditions allowed the continuous production of barley and wheat single cells (Figure 1). We showed that these cells were viable (Figure 2), kept their "identity" as they produced the P5CR proteins with the predicted MW (Figure 5) and were robust enough to remain alive in 24 well microtiter plate format for at least 10 days. These results suggested that the single cell suspension culture of wheat and barley could be an ideal platform to facilitate functional genomics of monocots. As our results showed, the multi-well plant cell suspension platform could provide a vehicle to study different elicitors on target gene expression under a range of environmental conditions. However, the real 'power' of the platform would be the creation of conditional mutant cells both somatic and those under going embryogenesis through the ability to target specific genes and gene product using siRNA for example. Outside of gene expression analysis the platform could be used in studies related to protein-protein interaction and gene product targeting. However, caution needs to be exercised in the extrapolation of the results obtained using isolated cells to the physiology of whole plants [26]. This issue is not unique to the proposed platform as, by definition, all experimental observations need to be verified to ratify the conclusions. Therefore we suggest the model system as a stepping stone towards a greater understanding of complex biological systems.

In order to characterise and further develop the system we choose to study the impact of selected abiotic stress elicitors. Barley is widely recognised as one of the most salt-tolerant crops [27] and exhibits higher tolerances of heat, drought, and osmotic stresses when compared to wheat [28]. Therefore while there is considerable variation within barley cultivars with respect to salt-tolerance, on the whole, barley cultivars are more salt-tolerant than the wheat cultivars in conditions of both normal and accelerated development [29].

In whole plant physiology it is widely reported that NaCl and PEG elicit an osmotic stress response [16,30]. Similar observations have been reported for plant cells cultures [31,32]. In plants, under osmotic stress, a range of compatibles solutes are produced, proline is one of these. It has been proposed that proline accumulation can serve as an adaptive mechanism to salt stress in higher plants [33]. Interestingly, although the reported plant response is increased proline production, the level of the produced proline is considerably different in different species and/or in sensitive and tolerant cultivars. Furthermore, it was reported that proline accumulation during osmotic stress is higher in sensitive than in the tolerant wheat genotypes [34] and this observation is in accordance with data reported for other species such as cassava [35]; two Mediterranean shrubs [36]; beech [37] and barley [38]. Therefore we could conclude, based on the reported general observations that the sensitive species respond to osmotic stress by accumulating higher levels of proline; furthermore, in the sensitive genotypes osmotic stress induced proline level are higher than in the tolerant one.

Given the documented role of proline as a compatible solute, we analysed the impact of osmotic stress on the viability of wheat and barley cells in relation to proline levels. At the whole plant level it is widely understood that barley exhibits greater tolerance to drought than wheat [28]. By studying the impact of osmotic stress on wheat and barley cells we had the opportunity to establish if the observed drought tolerance for barley versus wheat held true in culture. As described, the barley cultures did in fact exhibit greater tolerance to osmotic stress. The treatment of 100 mM NaCl reduced the number of viable cells over a 24 h period in wheat where as the barley cells did not exhibit any marked change (Figure 6). The fact that wheat and barley cells do appear to differ in their responses to osmotic stress and this response mimic the whole plant observation discussed above supports the rational that plant cells can be used as a model system.

The analysis of the proline levels in wheat and barley cells revealed that the wheat cells contained 3 times the level found in barley (Figure 7). When subject to osmotic stress the level of proline in both cell types increased over time, although there did not appear to be a correlation between concentration of NaCl and the level of proline, while under PEG treatment the response was slightly less in magnitude (Figure 7A and 7B). It is interesting to note that the amount of proline in wheat cell prior to treatment was 11 μg proline/10^6 ^live cells while for barley it was 3 μg proline/10^6 ^live cells. After exposure to 24 h of 100 mM NaCl treatment the level of proline in wheat had reached 19 μg proline/10^6 ^live cells while for barley the maximum observed was 4 μg proline/10^6 ^live cells, not very different from the initial level observed in wheat (Figure 7).

As stated in a variety of plants, stresses such as cold, heat, salt, drought, UV, and heavy metals significantly increase endogenous proline concentrations [39-41] and our results would seem to bear this out. However the wheat cells die with time, while the barley cells do not, when in both lines the level of proline increases in line with the widely documented response to stress (Figure 6 and 7).

As discussed above, P5CS and P5CR are two enzymes central to proline biosynthesis. Using RT PCR, normalised to tubulin, it was possible detect changes in the steady state level of P5CS and P5CR transcripts. In the wheat cells exposed to stress the level of the P5CS and P5CR transcripts appeared to increase over time (Figure 8). This correlates with the observed increase in total free proline content (Figure 7A). However the same analysis conducted for stressed barley cells did not illustrate the same transcript response in fact the trend, although not significant, is for a slight reduction in transcript level with time (Figure 9). Closer analysis of the data reveals that, in barley, both P5CS and P5CR exhibit a higher steady state level of transcript compared to wheat. At first sight this appears to contradictory, as the proline level in the barley without stress is significantly lower than in wheat. Moreover when stressed we observed an increase in proline, but not the transcripts associated with genes thought to be directly responsible for proline biosynthesis. Given these observations, it is reasonable to speculate that there are other levels of control being exerted in the barley cells. Proline can and does act as antioxidant mopping up reactive oxygen species (ROS) [42] and given that one common feature of stress is the production of ROS we could speculated that proline, having quenched the reactive oxygen species [43], it needs to be recycled out of the system, thereby preventing 'oxidised' proline toxicity. In response to abiotic stress, and during the recovery from it, the existence and the importance of P5C-Pro cycle was described recently [44]. The P5C-Pro cycle involves a balance between P5CR and the catabolic pathway activities involving the consecutive action of proline dehydrogenase that produces P5C and P5C dehydrogenase (P5CDH) that oxidizes P5C to glutamate. Hyperactivity of the cycle could generate mitochondrial ROS by delivering electrons to O_2 _[44].

**Figure 10:**
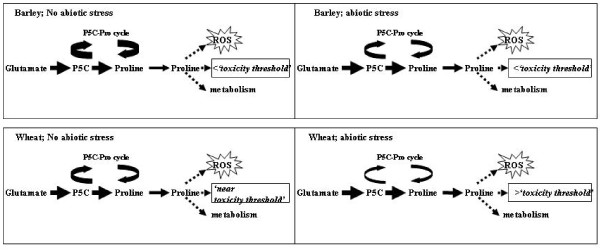
**The proposed model to explain the obtained differences in stress tolerance between wheat and barley suspension culture**. Diagrammatic model proposed to integrate the results obtained with the those reported in the literature to account for the differential response of wheat and barley to abiotic stress: For wheat under non-stressed conditions the high steady state level of proline is maintained by low rate of turnover/recycling activity of Pox, the net result is proline levels in wheat are close to a hypothetical 'threshold'. While in barley Pox activity is higher resulting in lower steady state levels of proline, which is below the 'threshold'. Under abiotic stress the activity of Pox is reduced and P5CS and P5CR genes are up regulated and proline increases in both wheat and barley, although for barley the proline level remains below the 'threshold and therefore does not suffer proline toxicity and tolerates the stress more efficiently. The colour intensity and thickness of the arrows represent the expression level differences in the different part of the pathways.

The emerging hypothesis based on our results combined with the accumulated knowledge of the role of proline in the plant cell is illustrated graphically as a model in Figure 10. The proposed diagrammatic model integrates our results with those reported in the literature to account for the differential response of wheat and barley to abiotic stress: For wheat under non-stressed conditions the high steady state level of proline is maintained by low rate of turnover/recycling activity of the P5C-Pro cycle, the net result is proline levels in wheat are close to a hypothetical 'threshold'. While in barley P5C-Pro cycle activity is higher resulting in lower steady state levels of proline, which is below the 'threshold'. Under abiotic stress the activity of P5C-Pro cycle is reduced and P5CS and P5CR genes are up regulated and proline increases in both wheat and barley, although for barley the proline level remains below the 'threshold and therefore does not suffer proline toxicity and tolerates the stress more efficiently. Work to verify the hypothesis generated as part of this work is currently underway in our laboratory.

## Conclusions

We have reported the development of single cell suspension of both wheat and barley and demonstrated the utility of cells in a multi-well format. The osmotic stress experiments using wheat and barley cell suspension cultures showed that barley was more tolerant to the applied osmotic stresses than wheat, which is in good correlation of the reported better tolerance of barley plant than wheat for osmotic and drought stresses (REF). The proposed model (Figure 10) could offer a plausible explanation as to why barley exhibits greater tolerance to osmotic stress than wheat. Therefore to conclude our results seem to indicate the potential of the wheat and barley cell suspension system as model to assist the pursuit of candidate genes that will help the crop development programmes through the implement of knowledge gained from functional genomics.

## Authors' contributions

DJ maintained the suspension cultures, carried out the cell viability, osmotic stress and RT-PCR experiments and evaluated the data. SB participated in the planning the experiments and evaluating the data, performed the FDA studies and was involved writing the article. EV participated in the planning the experiments and evaluating the data, performed the FDA studies and Western blot analysis and was involved writing the article. All authors read and approved the final manuscript.
